# Etiological Bacterial Spectrum of Purulent Inflammation of Cervical Lymph Nodes in Children With Unknown Causes of Lymphadenopathy

**DOI:** 10.7759/cureus.46276

**Published:** 2023-09-30

**Authors:** Yanko G Yankov

**Affiliations:** 1 Department of General and Operative Surgery, Medical University of Varna "Prof. Dr. Paraskev Stoyanov", Varna, BGR

**Keywords:** lymphadenopathy, children, maxillofacial surgery, oral surgery, neck, lymph node, purulent inflammation, bacteria, phlegmon, abscess

## Abstract

In the management of the non-specific purulent inflammation of the cervical lymph nodes in children, not only surgical treatment to evacuate the available pus but also antibacterial therapy is mandatory. Knowledge of the spectrum of the bacterial causative agents of this disease is of fundamental importance for it.

This retrospective study included 66 patients with purulent inflammation of the cervical lymph nodes with a mean age of 5.79 years, ranging from 29 days to 17 years, who were hospitalized for the period 2015-2022 in the three pediatric clinics at the St. Marina University Multispecialty Hospital for Active Treatment in Varna, Bulgaria, and operated by an oral or maxillofacial surgeon, in which for microbiological examination material from the suppurated lymph node was taken and analyzed first by direct microscopy for gram-positivity and then on a biochemical machine identifier VITEK (bioMérieux, Marcy-l'Étoile, France). In all of them, the patients and their parents did not provide anamnestic data explaining the etiology of their infectious diseases of the cervical lymph nodes. There was no clear cause that would explain the occurrence of cervical lymphadenopathy - skin infections, dental diseases, diseases of the ears and upper and lower respiratory tracts, onco-hematological diseases, and others. During the medical examination by pediatricians and surgeons, no entrances to the infection were found, but only local signs of inflammation (redness, swelling, and pain), increased levels of blood markers of inflammation (leukocytes, neutrophils, erythrocyte sedimentation rate, and C-reactive protein) and there was imaging evidence (ultrasound, magnetic resonance imaging, and computed tomography) of a purulent collection in the lymph nodes and/or in the soft tissues surrounding them. Exudate without microbial growth was found in 22 of the cases. The main causative agents of purulent lymphadenopathy are gram-positive bacteria (n=32, 72,73%) - *Staphylococcus aureus* (n=16), *Staphylococcus haemolyticus* (n=8), Beta-hemolytic streptococcus (n=2), and gram-positive mixed resident microflora (n=6). However, in 27.27% (n=12) of all 44 patients described in this article with isolated pathogens, cultures were gram-negative. These are *Bartonella henselae* (n=4), *Klebsiella pneumoniae* (n=4), *Klebsiella oxytoca* (n=2), and *Flavimonas oryzihabitans *(n=2).

## Introduction

Cervical lymphadenomegaly in children is a common condition. In healthy children, its frequency reaches up to 45%, and large, palpable lymph nodes are normally found in most of the superficial lymph basins - most often cervical, axillary, and inguinal, which, however, are not pathological [1-3]. In children, there is a progressive increase in lymphoid mass from birth to early adolescence, after which this lymphoid tissue gradually decreases during puberty [2,3]. Many lymph nodes are palpable in children, and in general, cervical nodes less than 20 mm, axillary nodes less than 10 mm, and inguinal nodes less than 15 mm are considered physiologic in pediatric individuals, whereas palpable epitrochlear and supraclavicular nodes should be viewed with suspicion and to be monitored and investigated [2]. Most researchers accept as the pathological limit of the size of the lymph nodes those whose diameter is greater than 10 mm. According to one of the largest studies of children with cervical lymph node disease, including 2687 patients from five different countries, in two-thirds of them (n=1822, 67.8%), the etiology remains unclear [1]. The case of the patients we present in this article is similar, but we are examining and analyzing children with an unknown etiological bacterial agent and an unknown gateway to infection.

## Materials and methods

This retrospective study included 66 patients with purulent inflammation of the cervical lymph nodes with a mean age of 5.79 years, ranging from 29 days to 17 years, who were hospitalized for the period 2015-2022 in the three pediatric clinics at the St. Marina University Multispecialty Hospital for Active Treatment in Varna, Bulgaria. In all of them, there was no clear cause that would explain the occurrence of cervical lymphadenopathy - skin infections, dental diseases, diseases of the ears, upper and lower respiratory tracts, onco-hematological diseases, and others. Only local signs of inflammation such as redness, swelling, and tenderness, imaging data (ultrasound, magnetic resonance imaging, and computed tomography), and elevated blood markers of inflammation (leukocytes, neutrophils, erythrocyte sedimentation rate (ESR) and C-reactive protein (CRP)) were observed. The operative intervention was performed under general anesthesia by specialists in oral and maxillofacial surgery from the Clinic of Maxillofacial Surgery at the same medical institution. The hospital stay was from three to five days, after which they were discharged fully recovered and without complaints. During the operative intervention, after an incision of the skin and subcutaneous tissue in the area of the most pronounced fluctuation and dissection of soft tissues, an enlarged lymph node or a bundle of those with exudative purulent inflammation was reached, and the existing pus was evacuated. In all, material was taken for microbiological examination, which was processed in the Clinic of Microbiology of the same medical institution - first by direct microscopy for gram-positivity and then on a biochemical machine identifier VITEK (bioMérieux, Marcy-l'Étoile, France). All children received antibacterial treatment prescribed by pediatricians in the appropriate doses according to their body weight.

## Results

**Figure 1:**
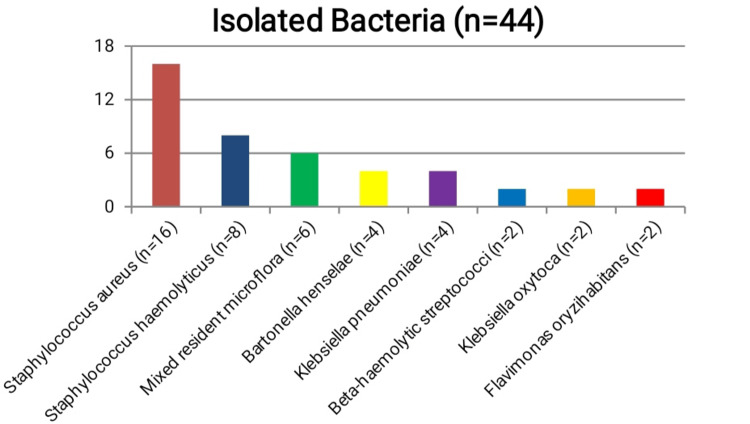
Schematic representation of the distribution of the isolated bacteria in the purulent inflammation of the cervical lymph nodes in the studied children

In 22 of all 66 examined children, no microbiological pathogens were isolated, i.e., cultures remained sterile (n=22). Of the remaining 44 samples in which microbial pathogens were isolated, the largest share was *Staphylococci *(n=24), which consisted of *Staphylococcus aureus* (n=16) and *Staphylococcus haemoliticus* (n=8). The following are gram-positive mixed resident microflora, which is represented by more than one bacterial species (n=6), *Bartonella henselae* (n=4), *Klebsiella pneumoniae* (n=4), beta-hemolytic streptococci(n=2), *Klebsiella oxytoca* (n=2) and *Flavimonas oryzihabitans* (n=2) (Table 1, Figure 1).

**Table 1:** Distribution of the isolated pathogens in the studied population (n=66)

Number by order	Isolated bacteria	Gram positive (+) or negative (-)	Number of isolates (n)
1	Staphylococcus aureus	+	16
2	Staphylococcus haemolyticus	+	8
3	Gram-positive mixed resident microflora	+	6
4	Bartonella henselae	-	4
5	Klebsiella pneumoniae	-	4
6	Beta-hemolytic streptococci	+	2
7	Klebsiella oxytoca	-	2
8	Flavimonas oryzihabitans	-	2
9	Exudate without microbial growth		22

## Discussion

Acute lymphadenitis, with a strong virulence of the pathogen causing it and a weakened immunity of the macroorganism, if not treated, passes through three successive stages - from serous inflammation of the lymph node; through purulent infection of the latter, in which the capsule of the lymph node is preserved, and the pus is limited in its internal state (suppurative, purulent inflammation of the lymph node); to the stage of lymphadenophlegmon, in which the capsule of the affected lymph node ruptures and the pus comes out of it into the adjacent soft tissues (abscess or phlegmon of lymphogenic origin). While in the first stage, the treatment is conservative with antibacterial preparations, in the remaining two purulent stages, it is mandatory to carry out surgical treatment in the form of incision, lavage, and drainage in order to evacuate the formed pus, accompanied by the application of antibacterial agents. The 66 cases described in this article are of children with diseases of the lymph nodes from the last two stages of development of their infectious inflammation (purulent inflammation of the lymph nodes and abscesses or phlegmons of lymphogenic origin).

Most often, suppurative lymphadenitis occurs in patients with reduced congenital or acquired immune forces [4,5]. The main causative agents of the disease are the gram-positive bacteria *Staphylococcus aureus*, *Streptococcus pyogenes*, and *Mycobacterium tuberculosis *[5]. Extremely rarely, the causative agents are gram-negative [5]. The present study is interesting because 12 out of 44 samples with isolated pathogens, which constitute 27.27% of all cases, were gram-negative bacteria. These are *Bartonella henselae *(n=4), *Klebsiella pneumoniae* (n=4), *Klebsiella oxytoca* (n=2), and *Flavimonas oryzihabitans* (n=2).

Most likely, the reasons for not isolating microbes in part of the samples (n=22) are incorrect collection of the microbiological material, which is most often expressed in the immersion of the sterile swab in the core of the pus, where there are mainly necrotic tissues and bacteria are absent, not immersing the swab in the nutrient medium of the container, improper storage of the sample at an inappropriate temperature, open lid of the sterile container or improper transportation.

Staphylococcus aureus is one of the most frequently isolated bacterial species in skin infections in both adults and children [3,6]. There is no literature data on its high frequency in inflammatory diseases of the lymph nodes in the neck region in adults and in children [1,3,6]. An article published in 1976 by Wald et al. described a case of a four-year-old child with a skin infection in which *Staphylococcus aureus* was isolated during drainage of a suppurated cervical lymph node [7]. There is no data on the isolation of the pathogen in children with no visible and explainable cause for their lymphadenopathy, both cervical and other localization, as in the cases described by us.

*Staphylococcus haemolyticus* is a part of the resident microflora in humans [8,9]. In adults, it occurs in a higher concentration in the hairy parts of the armpits and the pubic area, and in children, it is also found in the perineal area [10]. There is no literature data that it occurs in a higher frequency in the head and neck region, which would explain its isolation in such a large proportion (n=8) of the children examined in the present study. They are reported to inhabit the fur of placental mammals, including pets of this species [10], which may be one of the causes of purulent cervical lymphadenopathy in these children if they have had contact with them.

Gram-positive mixed resident microflora, containing more than one type of bacteria, is often found as a cause of abscesses and phlegmons in humans of any location [11,12]. Weakened local immune forces are the most common reason explaining this [4]. Therefore, it is not surprising that this type of causative agent occurs in such a large proportion of the examined children with purulent inflammation of the neck lymph nodes (n=6).

*Bartonella henselae* is the causative agent of cat scratch disease (CSD), also called felinosis. Regional lymphadenomegaly is typical for it in a significant percentage of sick children - according to some authors, up to 13.4% [13]. In all of them, however, a primary focus was discovered and described - a place from which the bacterium penetrated the macroorganism, i.e., the entrance point of the infection, which is most often a scratch or a bite from the animal [14]. Unusually, in the pediatric patients we describe, there were no entrance point marks on clinical examination, and there was no history from the children and their parents of being bitten or scratched by mammals. In all four children in whom *Bartonella henselae* was isolated, they and their parents denied any contact with cats in recent months. Given the fact that the microbiological examination is conclusive about the nature of the isolated pathogen, we assume that the children's contact with animals and an entrance point to the infection was there, and we suggest that *Bartonella henselae* should always be considered in the differential diagnosis of cervical lymphadenomegalia, including the purulent one, that requires surgical treatment.

*Klebsiella pneumoniae* is extremely rarely isolated from patients with purulent lymphadenitis of any localization [15,16]. In the world literature, there are only single cases of patients in which this bacterial species was found in adults with suppurated lymph nodes, but there are no reports of its isolation in children [17].

Beta-hemolytic streptococci, as representatives of the resident microflora in humans, are common pathogens in purulent diseases, including abscessed lymph nodes [18]. It is not uncommon for them to be isolated in the children we described.

*Klebsiella oxytoca* is a human commensal, but it is also an opportunistic pathogen living in the oral cavity, nose, and gastrointestinal tract [19,20]. It most commonly causes urinary tract infections and hemorrhagic colitis [19,20]. There is no evidence in the world medical literature of involvement of the lymph nodes by it, either in adults or in children, leading to their abscessing, as in the two cases described in the present report.

*Flavimonas oryzihabitans* is most commonly found in patients with a urethral catheter or a central venous or arterial line [21], those with a prolonged hospital stay or recent surgery (nosocomial infection) [22], and in individuals with accompanying diseases associated with causing immune deficiency (most often leukemias and HIV/AIDS) [23]. In neither of the two cases of children in whom the presence of this gram-negative bacterium was demonstrated in the suppurating cervical lymph nodes were present these accompanying diseases and circumstances that would explain the occurrence of this disease.

It is unusual for acute purulent inflammation of the lymph nodes, not only in children but also in adults, that their bacterial causative agents are of the gram-positive spectrum. In the world literature, only single cases of patients were described in which gram-negative microorganisms were isolated from patients with this pathology, while the present study found such representatives in a significant proportion of the examined children - 27.27% [24].

## Conclusions

The cause of purulent inflammatory diseases of the cervical lymph nodes in children cannot always be established, and the entry point of the infection cannot always be established, as in the 66 cases of children with purulent lymphadenopathy of the neck described in this article. In most cases, they are caused by gram-positive bacteria, but there are also situations in which the causative agents are gram-negative, as in 27.27% of the pediatric patients described in this literature work. This is of little importance for the surgical treatment of these patients, in which the present purulent exudate is evacuated, but it may be taken into consideration in the choice of antibacterial therapy, especially when it is prescribed empirically in such cases of emergency as those described in the present article.
